# Molecular Investigation of 2013 Dengue Fever Outbreak from Delhi, India

**DOI:** 10.1371/currents.outbreaks.0411252a8b82aa933f6540abb54a855f

**Published:** 2014-09-02

**Authors:** Nazia Afreen, Farah Deeba, Irshad Naqvi, Mohammad Shareef, Anwar Ahmed, Shobha Broor, Shama Parveen

**Affiliations:** Centre for Interdisciplinary Research in Basic Sciences, Jamia Millia Islamia, New Delhi, India; Centre for Interdisciplinary Research in Basic Sciences, Jamia Millia Islamia, New Delhi, India; Dr. M. A. Ansari Health Centre, Jamia Millia Islamia, New Delhi, India; Dr. M. A. Ansari Health Centre, Jamia Millia Islamia, New Delhi, India; Department of Biochemistry, College of Science, King Saud University, Riyadh, Kingdom of Saudi Arabia; Department of Microbiology, Jamia Hamdard, New Delhi, India; Centre for Interdisciplinary Research in Basic Sciences, Jamia Millia Islamia, New Delhi, India

## Abstract

Dengue fever is a self-limiting, acute febrile disease which may aggravate to haemorrhage, plasma leakage and organ impairment in small number of cases. An outbreak of dengue fever occurred in Delhi, India after rainy season in the year 2013. Dengue virus specific RT-PCR was carried out on 378 suspected blood samples that were collected during the outbreak. Dengue virus was detected in 71% samples with highest number of patients infected by DENV-2 (86%) followed by DENV-1 (19 %) and DENV-3 (8%). Co-infection with more than one DENV serotype was detected in 14% samples. Twenty nine DENV strains (10 DENV-1, 12 DENV-2 and 7 DENV-3) were sequenced for partial envelope protein gene. Phylogenetic analysis grouped DENV-1 strains in the American African genotype, DENV-2 strains in the Cosmopolitan genotype and DENV-3 in Genotype III. We report the serotype distribution, circulating genotypes and partial envelope protein gene sequence of 29 DENV strains detected during 2013 outbreak in Delhi, India.

## Introduction

Delhi, the capital of India, is a large metropolitan city which attracts thousands of people from all over India for better business and career opportunities. Many housing areas have cropped up to meet the demands of its ever growing population but the infrastructure and drainage facilities are poor causing favorable conditions for mosquito breeding. As a result, Dengue fever, a mosquito borne disease, is common in Delhi reaching epidemic proportions after every 3-4 years. Dengue fever outbreaks have been reported in Delhi in 1967^1^ , 1970^2^ , 1982^3^ , 1988^4^ , 1996^5^ , 2003^6^ , 2006^7^ and 2010^8^. Dengue fever is characterized by fever, aches and pain, rash, nausea, vomiting, weakness and prostration while in some cases it aggravates to haemorrhage, plasma leakage and organ impairment^9^. A vaccine for dengue is not available yet as the virus exists as 4 serotypes which do not cross-protect and enhance the risk of severe disease by antibody dependent enhancement of infection^10^. There was an increase in the number of cases of dengue fever reported from Delhi and all over India in the year 2013^11^. The present study was undertaken to characterize dengue viruses detected during the outbreak in Delhi in 2013.

## Materials and Methods

The study was granted approval by Institutional Ethics Committee, Jamia Millia Islamia and was done in accordance with the World Medical Association Declaration of Helsinki. Acute phase blood samples were collected from suspected dengue patients from Dr. M. A. Ansari Health Centre, Jamia Millia Islamia, New Delhi. Dr. M. A. Ansari Health Centre is a small health centre situated in the University campus which provides basic medical facilities to approximately 40,000 university students, employees and their dependents. Patients showing symptoms of fever accompanied by any two of the following; nausea, vomiting, rash, aches and pains, positive tourniquet test, leukopenia, were enrolled in the study. Written informed consent in English and Hindi was obtained from all adult subjects or a parent or guardian in case of minors. The age/sex, clinical symptoms and duration of fever of the patients were recorded in proformas. Serum was separated from the blood samples and stored at-80°C in aliquots. Dengue viruses were detected in the serum samples by semi nested RT-PCR for C-prM region as described previously^12^. RNA was extracted from the serum samples using QIAamp Viral RNA Mini kit (Qiagen, Germany) according to the manufacturer’s instructions. Extracted RNA was aliquoted and stored at -80°C. RNA was reverse transcribed into complimentary DNA (cDNA) copy in a 25µL reverse transcription (RT) reaction mixture using 20ng of random primers (Promega, USA), 1mM dNTPs (Promega, USA), 8U of rRNAsin (Promega, USA) and 10U of Avian Myeloblastosis Virus reverse transcriptase (Bangalore Genei, India) and 13.75µL RNA. The RT reaction mixture was incubated at 37°C for 90 minutes followed by enzyme inactivation at 65°C for 15 minutes. First round (external) PCR was carried out in a 25µL reaction using 1X Taq buffer, 200µM dNTPs (Promega, USA), 0.4µM primers (D1 and D2)^12^, 1.5U of Taq DNA polymerase (Bangalore Genei, India) and 3 µL cDNA. The PCR was carried out as follows: 94°C for 2 minutes followed by 35 cycles of 94°C for 30s, 52°C for 30s and 72°C for 60s and a final extension at 72°C for 10 minutes. A 511bp segment specific to all 4 dengue serotypes was amplified in this external PCR.

External PCR was followed by semi nested PCR using D1 primer and 4 serotype-specific primers; TS1, TS2, TS3 and TS4^12^. Nested PCR was carried out in a 25µL reaction using, 1X Taq buffer, 200µM dNTPs (Promega, USA), 0.4µM primers (D1, TS1, TS2, TS3 and TS4), 1.5U of Taq DNA polymerase (Bangalore Genei, India) and 1 µL of the diluted (1:5) external PCR product. The nested PCR was run as follows: 94°C for 1 minute followed by 25 cycles of 94°C for 30s, 54°C for 30s and 72°C for 60s and a final extension at 72°C for 10 minutes. The sizes of the nested PCR products were specific for each dengue serotype i.e. 482-bp for DENV-1, 119-bp for DENV-2, 290-bp, for DENV-3 and 392-bp for DENV-4. PCR products were electrophoresed through 2% agarose gel, stained with ethidium bromide and examined under ultraviolet light using a gel documentation system (Wealtec, USA). Singleplex nested PCR reactions with D1 and one of the type specific primers (as applicable) were carried out for confirmation of the co-infection of serotypes using same conditions as mentioned above.

Partial segments (448bp, 574bp and 582 bp) of envelope protein gene were amplified using published primers for DENV-1^13^, DENV-2^14^ and DENV-3^15^ strains respectively. The respective amplicons were visualized on 2% agarose gel. The specific bands were cut from the gel and DNA was extracted from the gel using QIAquick Gel Extraction Kit (Qiagen, Germany). The amplicons were sequenced in both forward and reverse directions by commercial sequencing services (Xceleris Labs, India). The identity of the sequences were checked and confirmed by BLAST. The forward and reverse sequences were aligned and manually edited in Genedoc (v2.7.000) software to obtain the consensus sequence. The sequences obtained in the present study and other sequences retrieved from the GenBank, were aligned in ClustalW (2.1). Phylogenetic trees were constructed using Maximum Likelihood method in MEGA 6.06 software. Genetic distances were calculated using Tamura- Nei model of nucleotide substitution. The robustness of the tree was assessed with 1000 bootstrap replicates. Nauru strain (GenBank Accession Number: U88535) was used as prototype strain for DENV-1, NGC 44 strain (GenBank Accession Number: AF038403) for DENV-2 and H87 strain (GenBank Accession Number: M93130) for DENV-3.

## Results

A total of 378 acute phase blood samples were collected from suspected dengue patients attending the Out Patient Department (OPD) of Dr. M. A. Ansari Health Centre, Jamia Millia Islamia from 29 August, 2013 to 19 November, 2013. Patients included in the study showed symptoms of fever, headache, body-ache, nausea, vomiting and weakness. Information regarding age was available for 326 patients, regarding sex for 334 patients and regarding duration of illness for 315 patients. The duration of acute phase of illness ranged from 1-15 days (mean ± SD: 3.4± 2.4). The mean age of patients was 20.68 years (SD ± 12.3years) with male to female ratio of 1.8: 1. Dengue virus specific RT-PCR was conducted on all samples. DENV was detected in 269 (71.16%) samples out of 378 samples tested. The male to female ratio among the dengue virus positive samples was 1.59:1. DENV-2 was detected in highest number of patients (232, 86.24%) followed by DENV-1 (52, 19.4%) and DENV-3 (23, 8.58%) among the DENV positive samples. Co-infection with more than one DENV serotype was detected in 37 (13.75%) samples. Majority of concurrent infection samples were co-infected by DENV-1 and DENV-2 (n=26). Ten samples were co-infected by DENV-2 and DENV-3 and one sample was co-infected by DENV-1 and DENV-3. Singleplex nested PCR reactions with D1 and one of the type specific primers (as applicable) were carried out for all the concurrent infection samples and single specific bands were visualized on agarose gel.

Molecular characterization of DENV strains detected in the 2013 outbreak was also carried out by sequencing domain III of the envelope protein gene. E gene fragments of 10 DENV-1, 12 DENV-2 and 7 DENV-3 strains were sequenced in the present study. The sequences were deposited in the GenBank database with following accession numbers; GenBank: KJ729163-KJ729172 (DENV-1 strains) GenBank: KJ729151-KJ729162 (DENV-2 strains) and GenBank: KJ729173-KJ729179 (DENV-3 strains). Phylogenetic analysis of the DENV strains was carried out based upon the sequenced region. A 353 bp (117 amino acid) region of envelope genes of 46 DENV -1 strains including 10 DENV-1 strains sequenced in the present study were aligned. The aligned region spanned 1921-2273 bp of DENV-1 genome, 993-1345 bp of the E protein gene and 332-448 amino acid of the E protein (numbering based on the prototype). These strains grouped within the American African Genotype (Fig.1). Six mutations i.e. PheE339Ile, SerE341Thr, AlaE371Thr, Val E382Ile, Ile E438Val, Ile E441Val were detected in the study strains in comparison to the prototype strain. All these mutations have been previously reported. These study sequences showed nucleotide distance of up to 0.3% amongst themselves while amino acid sequences were identical. The study sequences showed nucleotide distance of 11.2-11.6% and amino acid distance of 5.3 % when compared to the prototype.

**Maximum Likelihood phylogenetic tree of Denv-1 strains:**
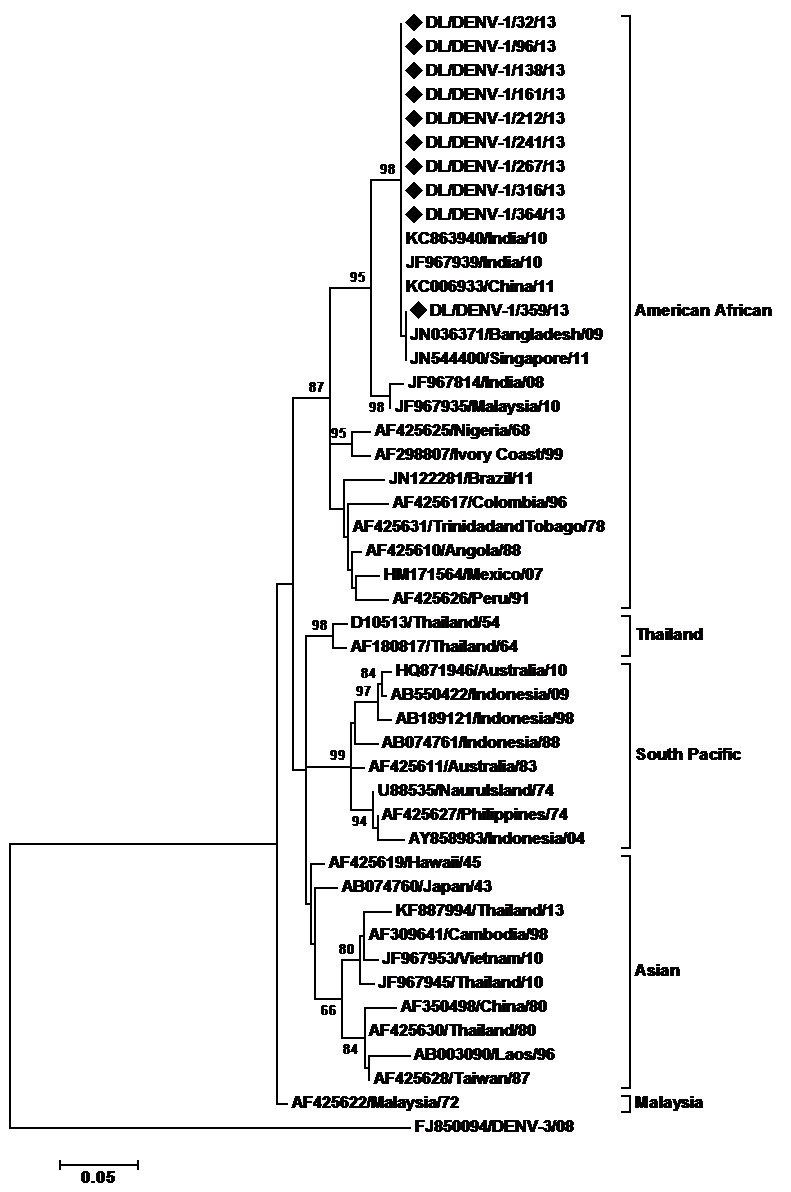
The tree is based upon partial E protein gene sequences. The sequences obtained in the study are marked by black diamonds. Other strains are represented by their GenBank accession number followed by country of origin and last two digits of year of isolation. The numbers on nodes represent bootstrap values generated by 1000 replications. Bootstrap values of >65 are shown. The branch lengths are proportional to the number of nucleotide changes as indicated by the scale bar (0.05 substitutions per site). The tree is rooted by DENV-3 strain.

For DENV-2 strains, a 449bp (149aa) region of E gene of 45 strains including 12 strains sequenced in the present study was aligned. The region corresponded to 1801-2249 bp of the DENV-2 genome, 865-1313 bp of the E protein gene and 289-437 amino acid of the E protein (numbering based on the prototype). The DENV- 2 strains of the present study clustered within the Cosmopolitan genotype (Fig. 2). The study strains showed nucleotide distance of up to 3% whereas amino acid sequences showed distances of up to 0.7%. Nucleotide distance of 5.8 to 6.3% and amino acid distance of 1.4-2% were identified when compared to the prototype strain. Two mutations, AsnE390Ser and IleE402Phe were detected in the study strains as compared to the prototype strain which are all previously reported.

**Maximum Likelihood phylogenetic tree of Denv-2 strains:**
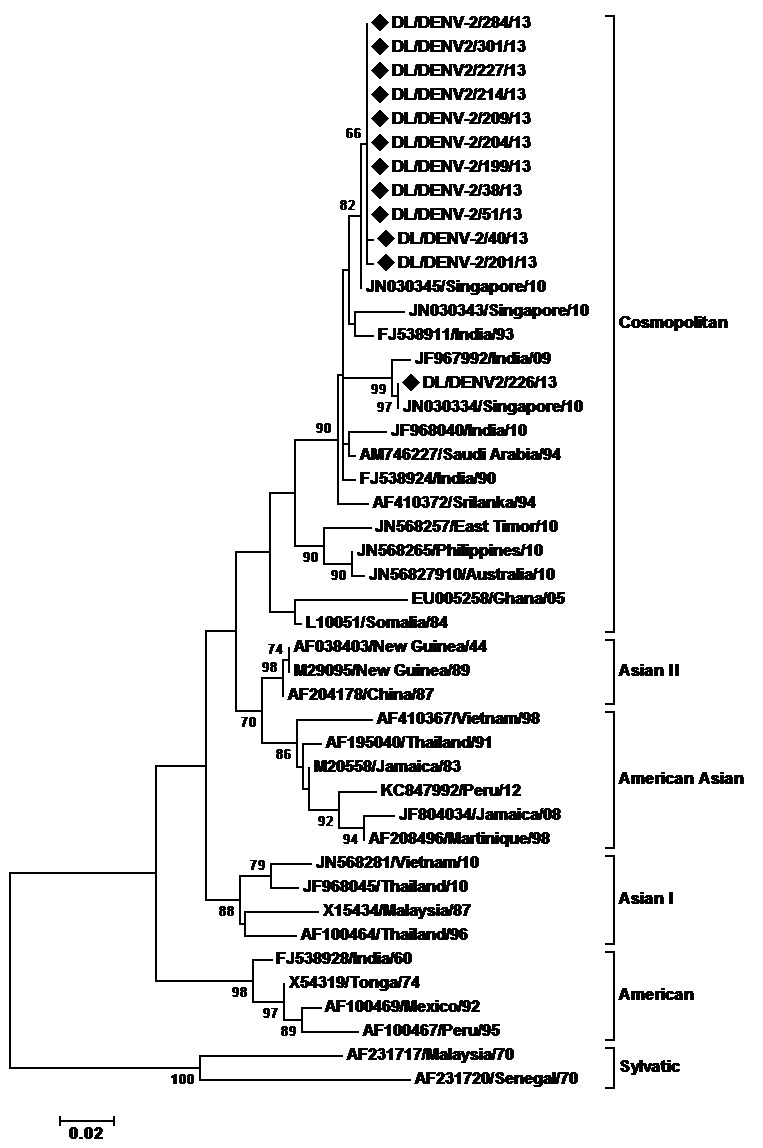
The tree is based upon partial E protein gene sequences. The sequences obtained in the study are marked by black diamonds. Other strains are represented by their GenBank accession number followed by country of origin and last two digits of year of isolation. The numbers on nodes represent bootstrap values generated by 1000 replications. Bootstrap values of >65 are shown. The branch lengths are proportional to the number of nucleotide changes as indicated by the scale bar (0.02 substitutions per site). The tree is rooted by the sylvatic DENV-2 strains.

Phylogenetic analysis of DENV-3 strains was carried out by aligning 485 bp (161 amino acid) region of E gene of 27 strains including 7 DENV-3 strains sequenced in the present study. The aligned region was from 1600 to 2084 bp of the DENV-3 genome, 666-1150 bp of the E protein gene and 223-383 amino acid of the E protein (numbering based on the prototype). The DENV- 3 strains of the present study grouped within the genotype III (Fig. 3). The DENV-3 strains showed nucleotide distances of up to 1.3% and amino acid distances of up to 0.6% among themselves. Nucleotide distance of 5.6 to 6.1% and amino acid distance of 3.2-3.8% were detected when compared to the prototype strain. Five mutations, all previously reported, were detected in the sequenced strains, LysE225Glu, ThrE270Asn, LysE291Glu, LeuE301Thr and LysE383Asn with reference to the prototype.

**Maximum Likelihood phylogenetic tree of DENV-3 strains:**
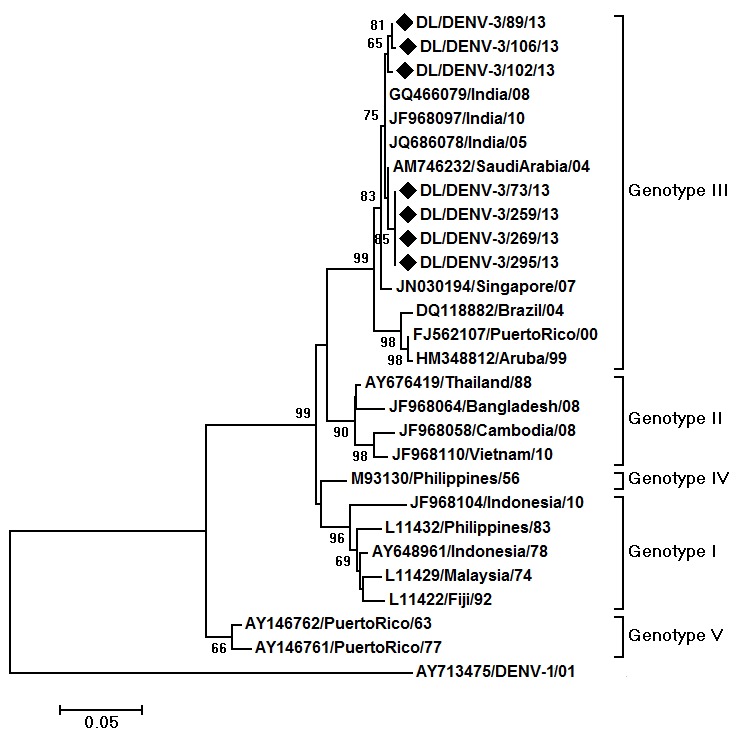
The tree is based upon partial E protein gene sequences. The sequences obtained in the study are marked by black diamonds. Other strains are represented by their GenBank accession number followed by country of origin and last two digits of year of isolation. The numbers on nodes represent bootstrap values generated by 1000 replications. Bootstrap values of >65 are shown. The branch lengths are proportional to the number of nucleotide changes as indicated by the scale bar (0.05 substitutions per site). The tree is rooted by DENV-1 strain.

Both serotypes detected in co-infected samples could not be sequenced for the E gene. Therefore, three co-infected samples were sequenced for C-prM gene junction. Two of such samples were co-infected by DENV-1 and 2 and one sample was co-infected by DENV-2 and 3. The sequences were confirmed by BLAST. These sequences were submitted to the GenBank with accession numbers KM114683- KM114685. The DENV-2 sequences were <200 bp and hence were not accepted by the GenBank (available upon request). As described by Chinnawirotpisan et al.^16^, the sequences were subjected to a preliminary phylogenetic analysis including prototype strain of all four dengue virus serotypes. Phylogenetic trees showed their similarity to their prototypic strains and not to the prototypic strain of other three serotypes confirming their identity (Fig.4).

**Maximum Likelihood phylogenetic tree based on C-prM gene region of A) DENV-1 strains B) DENV-2 strains C) DENV-3 strain:**
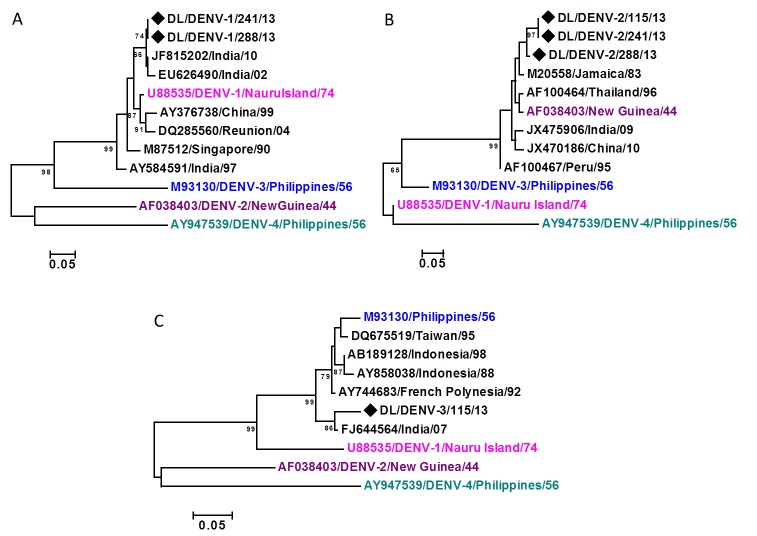
The sequences were obtained from co-infecting strains detected in the outbreak. The sequences obtained in the study are marked by black diamonds. Other strains are represented by their GenBank accession number followed by country of origin and last two digits of year of isolation. The numbers on nodes represent bootstrap values generated by 1000 replications. Bootstrap values of >65 are shown. The branch lengths are proportional to the number of nucleotide changes as indicated by the scale bar (0.05 substitutions per site). Prototype strains of all 4 dengue serotypes are included in the trees.

## Discussion

An outbreak of dengue fever occurred in Delhi after monsoon season in 2013. We have investigated the outbreak by conducting dengue virus specific RT-PCR on 378 suspected acute phase blood samples. Dengue viruses were detected in 71% of the samples confirming that the fever outbreak was caused by dengue viruses. The outbreak was mainly caused by DENV-2 serotype viruses. Dengue fever outbreaks are generally dominated by a particular serotype as reported from different parts of the world. DENV-2 dominated fever outbreaks were reported from Africa in 2007 and again in 2010^17^. DENV-3 dominated outbreaks have been reported in 2013 in Solomon Islands^18^, in 2009-2010 from Nicaragua^19^ and in 2004 from Indonesia^20^. Dengue type 1 dominated outbreak was reported in 2001 from Myanmar^21^. A dengue 4 dominated outbreak was reported in 2009 in French Polynesia^22^.

Three dengue virus serotypes were detected in the present study. DENV-2 dominated the outbreak with DENV-1 and DENV-3 being detected in small number of samples. DENV-4 was not detected in our study. This is the first report of molecular detection of dengue viruses during 2013 dengue fever outbreak from Delhi, India. Chakravarti and colleagues^23^ had reported detection of all 4 serotypes of dengue during 2007-2009 in Delhi. Another recent study has reported a DENV-2 dominated outbreak in Orissa state of India in 2010-2011 in which 72% samples were detected positive by RT-PCR and IgM ELISA^24^. A DENV-2 dominated outbreak was earlier reported in the year 1996^25^ in Delhi. All four serotypes were detected without any dominance by a particular serotype in 2003 outbreak in Delhi^26^ whereas DENV-3 was found to be the dominant serotype in the 2006 dengue outbreak^27^.The last dengue epidemic occurred in Delhi in 2010 in which 12.5% samples were detected positive for dengue viruses by RT-PCR and DENV-1 serotype was detected in 63% samples^28^. Our results show a change in serotype dominance from Dengue virus type 1 in 2010 to dengue virus type 2 in 2013 in Delhi.

Co-infection with more than one dengue virus serotypes was also detected (14%) in the present investigation. Bharaj et al had reported concurrent infection in 19% of the positive samples in 2006 in Delhi^27^. A very high percentage of co-infection by different serotypes (56.8%) was reported from Kerala, Southern state of India in 2008^29^ . DENV-1 strains of American African genotype, DENV-2 strains of the Cosmopolitan genotype and DENV-3 strains of Genotype III were detected in the 2013 outbreak.. The genotypes reported in the present study are known to be circulating in India in recent years (Afreen, unpublished data,^23^^,^ 30 ^,^ 31). Alteration in circulating DENV-1, 2 and 3 genotypes was not observed in the year 2013. This may be due to complete dominance of these genotypes over any other genotype which may have been imported but failed to establish. Another factor could be a selective advantage of these genotypes in Indian hosts (both human and mosquito) or in Indian climate.

## Conclusions

Molecular detection and serotyping of dengue viruses were carried out on acute phase blood samples that were collected during 2013 dengue fever outbreak from Delhi. Dengue viruses were detected in more than two third of the samples. Dengue virus serotype 2 was detected as the dominant serotype in the outbreak. Co-infection by different serotypes was also detected in the outbreak. Twenty nine outbreak strains (10 DENV-1, 12 DENV-2 and 7 DENV-3) were sequenced for partial envelope gene. Phylogenetic analysis grouped DENV-1 strains in the American African genotype, DENV-2 strains in the Cosmopolitan genotype and DENV-3 in Genotype III. The study will be helpful in the study of the epidemiology of dengue fever.

## Competing Interests

The authors have declared that no competing interests exist.
